# Supplementary material for: Symmetry-based reciprocity: evolutionary constraints on a proximate mechanism
Source: PeerJ. 2016 Mar 15;4:e1812. doi: 10.7717/peerj.1812 (PMC4797768; doi:10.7717/peerj.1812)
Supplement: Supplemental Information 2 — Supplementary methods and results. [file peerj-04-1812-s002.pdf]

Supplementary Material for:

Symmetry-Based Reciprocity: Evolutionary Constraints on a Proximate Mechanism

by

Marco Campennì and Gabriele Schino

SUPPLEMENTARY METHODS

Model Description

The model description offered below follows the standardized ODD (Overview, Design concepts, Details) protocol for describing individual and agent based models (Grimm et al., 2006).

Purpose

We developed two agent based models of symmetry-based reciprocity (one relying on an arbitrary tag and the other on inter-individual proximity) and tested their ability both to reproduce significant emergent features of cooperation in group living animals and to promote the evolution of cooperation.

State Variables and Scales

In these models time is represented discretely. In the first set of models (Tag models) space is not explicitly modeled; in the second set of models (Proximity models) space is explicitly modeled. During each time period, agents execute the commands described below (see the Process Overview and Scheduling).

Process Overview and Scheduling

These models proceed in discrete time steps, and entities execute procedures according to the following ordering:

Tag model:

- Interact: an agent (the "actor") is randomly selected from the whole population of N agents; then, a subset of other agents (the "candidates") is randomly extracted among the remaining agents

- Exchange cooperation following a specific rule, in this case, social tag difference: the actor compares its own tag with the tags of the candidates, and directs its cooperative behavior towards the candidate whose tag is the most similar to its own (that is, the actor calculates the absolute differences between its own and the candidates' tags and chose the candidate with the smallest absolute difference)

Proximity model:

- Move: an actor is randomly selected; it then selects a direction of travel randomly, and moves one unit length, i.e., it follows a brownian random-walk
- Interact: a subset of candidates is randomly extracted among the remaining agents
- Exchange cooperation following a specific rule, in this case, spatial proximity: the actor calculates the distance between itself and the candidates and directs its cooperative behavior towards the closest candidate

In multi-generations models there is an additional event occurring over time:

- Reproduction and selection: a subpopulation of worst performing agents (20% of the whole population) is removed from the population. In order to keep population size constant, a subpopulation of best performing agents (20% of the whole population) is made replicate themselves following a mutation rate and effect. The remaining 60% of the population has its tag initialized.

Design concepts

Basic Principles

While the evolution of reciprocal cooperation has attracted an enormous attention, the proximate mechanisms underlying the ability of animals to cooperate reciprocally are comparatively neglected. Symmetry-based reciprocity is a

hypothetical proximate mechanism that has been suggested to be widespread among cognitively unsophisticated animals. We developed two agent-based models of symmetry-based reciprocity (one relying on an arbitrary tag and the other on inter-individual proximity) and tested their ability both to reproduce significant emergent features of cooperation in group living animals and to promote the evolution of cooperation.

Emergence

In these models, populations formed by agents adopting symmetry-based reciprocity showed differentiated "social relationships" and a positive correlation between cooperation given and received, two common aspects of animal cooperation. However, when reproduction and selection across multiple generations were added to the models, agents adopting symmetry-based reciprocity were outcompeted by selfish agents that never cooperated.

Adaptation

Agents are characterized by simple properties and behaviors and they behave according to the rules defining their strategies (i.e., cooperators or selfish agents). In a first variant of the evolutionary models, agents adopt one of two different behavioral strategies, choosing cooperative or selfish. In a second variant of the evolutionary models, rather than having two discrete strategies, agents are characterized by an individual probability of behaving cooperatively.

Neither fixed nor mixed strategies vary over time: they are not affected by the outcome of past interactions or by environmental conditions and mutations operate modifying the offspring's strategy. In multi-generations models the agents' implicit

goal is to maximize their fitness, according to the simple fitness function that we defined (see Objectives below).

Objectives

Cooperation implied a cost for the actor and a benefit for the recipient. The fitness of each agent was calculated as the difference between the accumulated benefits received and costs incurred during a generation cycle. The selection process is described in the Process Overview and Scheduling.

Learning

No individual learning process is implemented in this model. Agents are not able to change their adaptive traits over time as a consequence of their experience.

Prediction

Agents in this model lack the ability to predict the outcome of future environmental variability or future social interactions. They do not integrate information across time periods.

Sensing

Agents decide to provide cooperation toward their interaction partners selecting the interactant minimizing an interaction function (i.e., social tag difference and spatial proximity, respectively). No memory is involved in the process.

Interaction

Agents interact by selecting an interaction partner among a set of potential partners.

Stochasticity

Individual social tag (for the Tag models) and coordinates in space (for the Proximity models) both have stochastic components: they are randomly initialized.

Observations

Reported data are averaged from 100 replicates and 30 replicates, for single generations models and multi-generations model, respectively. Simulations were run for 1000 ticks (i.e., time units or steps of the model) for single generation models and 50 (or 200) generations of 1000 ticks each for multiple-generations models.

Initialization

All runs were initialized according to parameters shown in Tables 1 and 2 in the main text.

SUPPLEMENTARY RESULTS

Some of the figures included in these supplementary results are also shown in the main text of the paper. They are reproduced here to facilitate comparison with the figures obtained using different combinations of parameters.

"Single-generation" models

For each combination of parameters, we obtained 100 matrices of cooperation exchanged between agents. Table S1 summarizes the social network values obtained for the Tag Model and the Proximity Model while varying the number of randomly selected candidates among which the agent could make its choice (the candidates). Table S2 summarizes the relations between cooperation given and received. Figures S1-S4 exemplify the effect of variation in the number of candidates on the networks of cooperation exchanged among agents and on the relations between cooperation given and received.

		Tag Model		Proximity model	
		Centralization index	Modularity	Centralization index	Modularity
**Number of candidates**	**2**	16.66±0.008	0.195±0.007	*7.31±0.018*	0.087±0.010
	**10**	13.33±0.017	0.578±0.005	16.34±0.038	0.322±0.024
	**25**	62.90±0.084	0.712±0.003	17.83±0.030	0.419±0.025
	**49**	97.81±0.0001	0.738±0.023	20.67±0.036	0.470±0.026

Table S1. Social network measures obtained in the "single-generation" models. Reported are means and standard deviations based on 100 simulations for each combination of parameters. All mean values obtained (except for the single value shown in italics) are within the range of values observed in primates, as reported by Pasquaretta et al. (2014).

		Tag Model	Proximity Model
**Number of candidates**	**2**	0.611±0.011	0.62±0.021
	**10**	0.951±0.003	0.921±0.010
	**25**	0.924±0.003	0.916±0.011
	**49**	0.663±0.002	0.861±0.019

Table S2. Results of within-subject linear regression between cooperation given and received. Reported are mean R-squared (and standard deviations) based on 100 simulations for each combination of parameters.

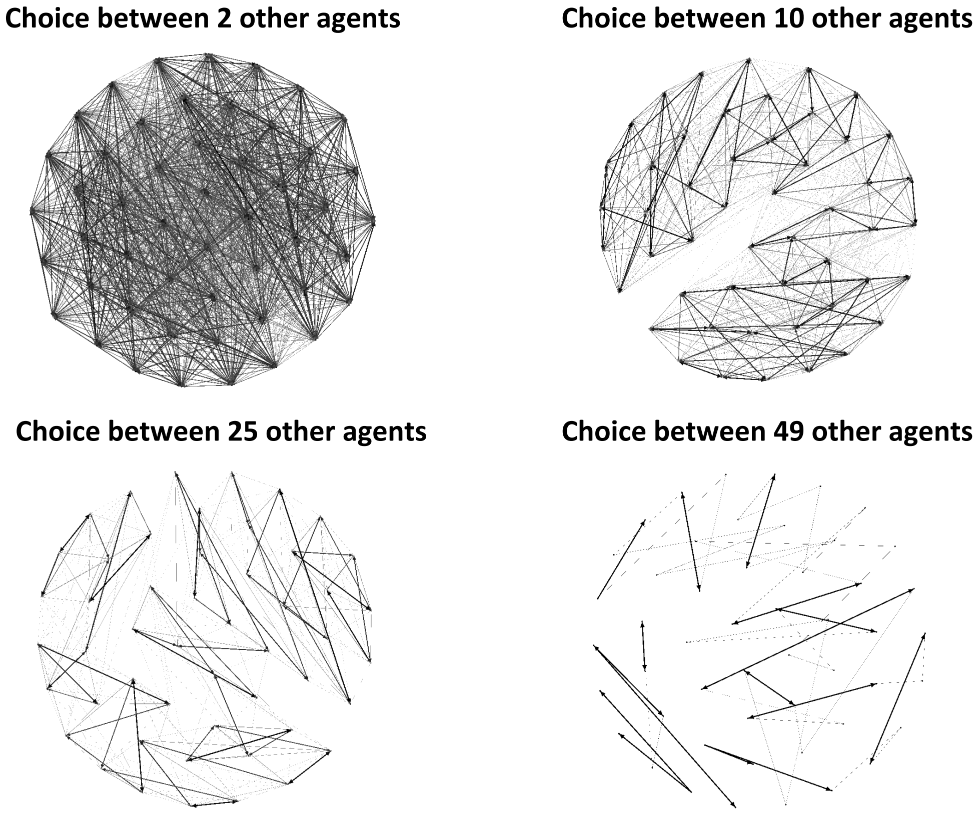

Figure S1. Representative distributions of cooperation among agents in the Tag Model. Networks of cooperation exchanged in populations of agents that differed in the constraints imposed on free choice (i.e., in the number of randomly selected candidates among which the agent could make its choice). Networks were visualized using Gephi version 0.8.2 adopting the Fruchterman-Reingold algorithm. Dots represent agents and the width of the connecting arrows is proportional to the amount of cooperation exchanged.

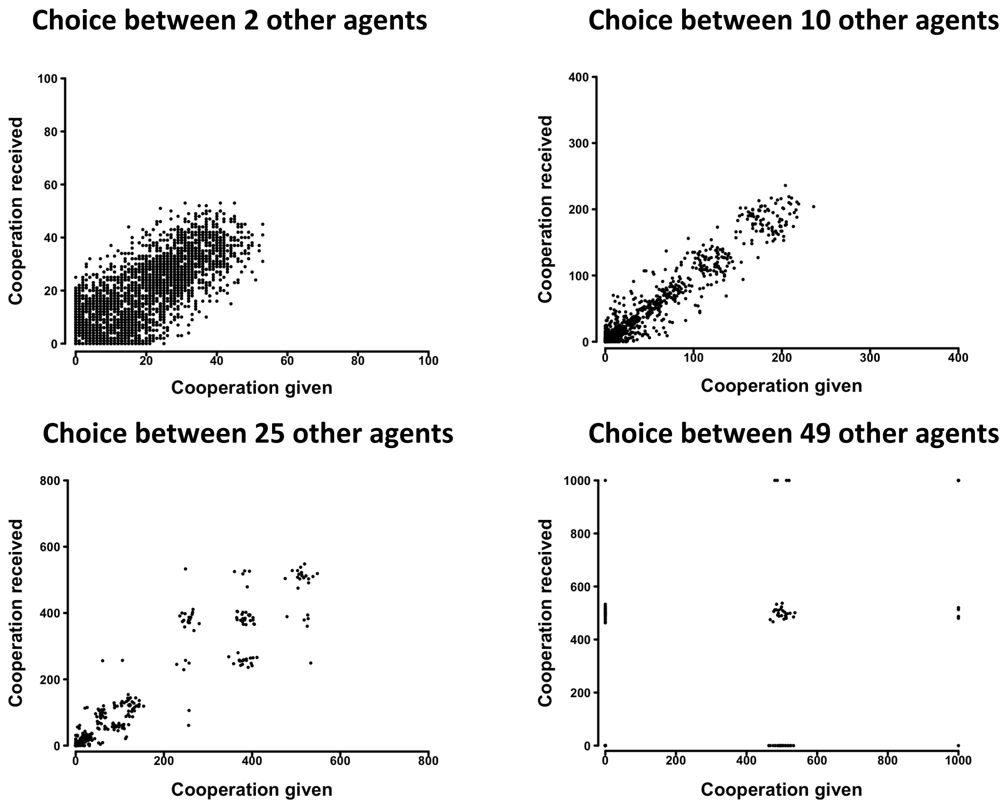

Figure S2. Representative relations between cooperation given and cooperation received in the Tag Model. Agents differed in the constraints imposed on free choice (i.e., in the number of randomly selected candidates among which the agent could make its choice). Each dot represents a dyad of agents. Note that in some of the panels (especially when choice was between 49 other agents) dots overlap extensively, so that fewer dots are visible. In fact, in all panels the number of dots (i.e., of dyads of agents) is the same.

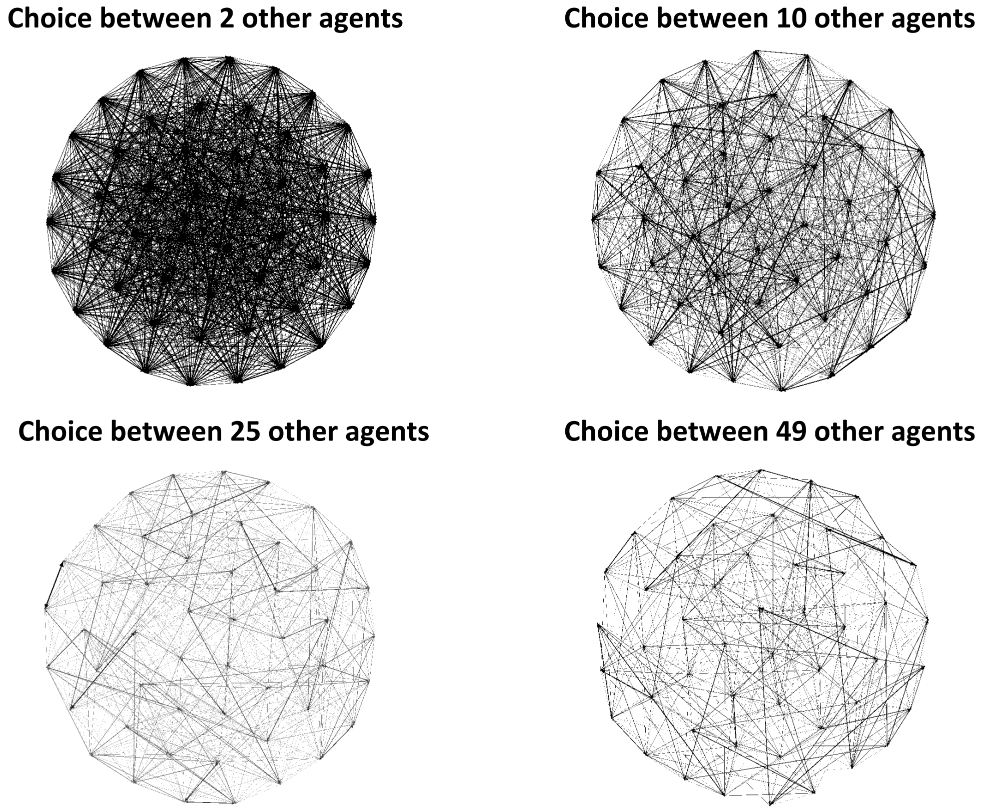

Figure S3. Representative distributions of cooperation among agents in the Proximity Model. Networks of cooperation exchanged in populations of agents that differed in the constraints imposed on free choice (i.e., in the number of randomly selected candidates among which the agent could make its choice). Networks were visualized using Gephi version 0.8.2 adopting the Fruchterman-Reingold algorithm. Dots represent agents and the width of the connecting arrows is proportional to the amount of cooperation exchanged.

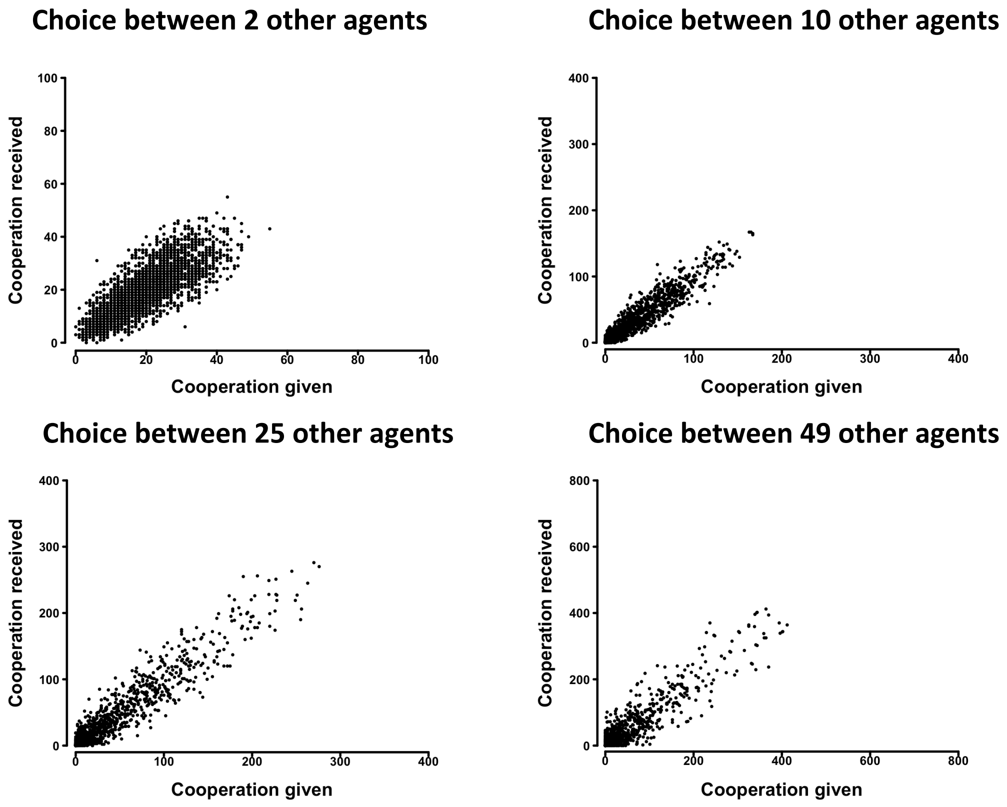

Figure S4. Representative relations between cooperation given and cooperation received in the Proximity Model. Agents differed in the constraints imposed on free choice (i.e., in the number of randomly selected candidates among which the agent could make its choice). Each dot represents a dyad of agents.

"Multi-generation" evolutionary models

"Two-strategy" models

Figures S5-S8 show the results of the simulations obtained running the "two-strategy" variant of the evolutionary Tag Model, where the relative success of two fixed strategies (symmetry-based cooperation and selfish) was evaluated along generations. In the different figures, agents could make their choice among 2, 10, 25 or 49 candidates.

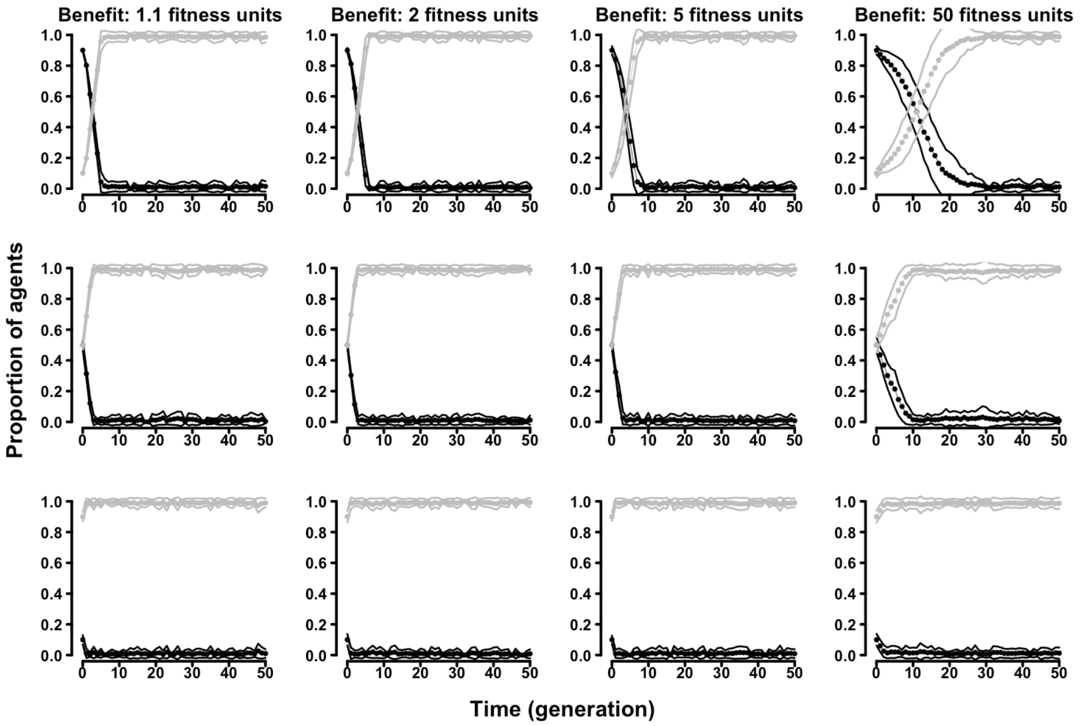

Figure S5. The evolution of cooperation in the "two-strategy" Tag Model. Populations were formed by agents adopting either of two different strategies: symmetry-based cooperation (black dots) and selfish (grey dots). Symmetry-based cooperators could make their choice among 2 randomly selected other agents. Populations varied in relation to their initial composition and in the fitness benefits of receiving cooperation. Cost of cooperation for the actor was always 1 fitness unit. Each panel shows the relative proportions of the two strategies along successive generations in 30 replicates for each combination of parameters (means and standard deviations).

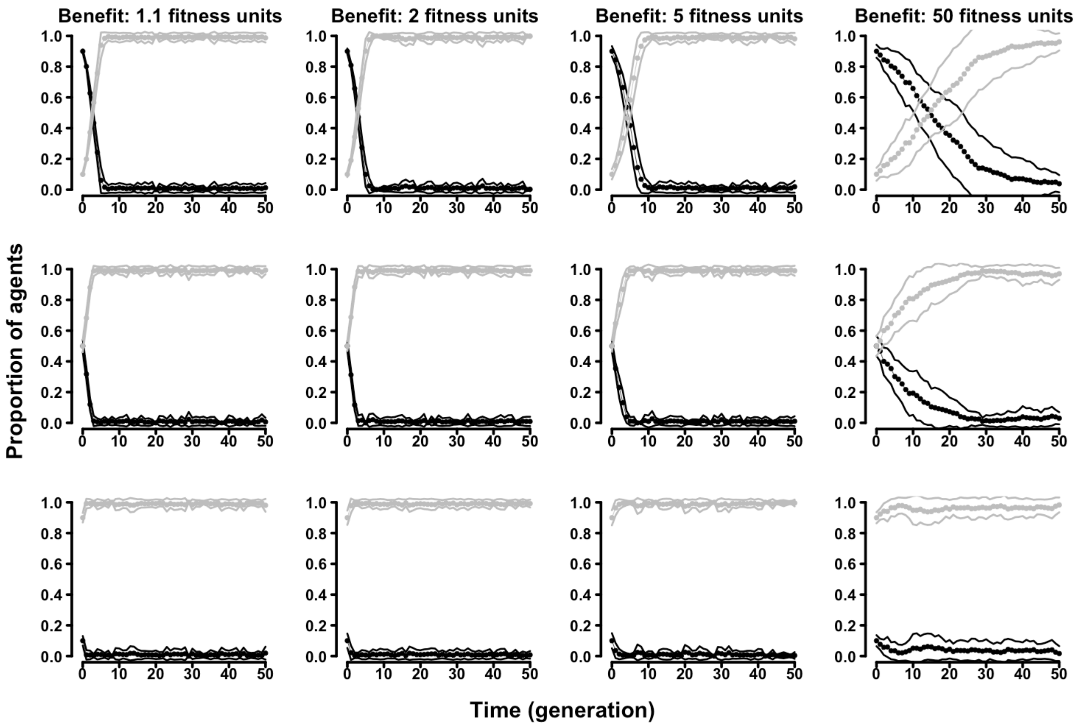

Figure S6. The evolution of cooperation in the "two-strategy" Tag Model. Populations were formed by agents adopting either of two different strategies: symmetry-based cooperation (black dots) and selfish (grey dots). Symmetry-based cooperators could make their choice among 10 randomly selected other agents. Populations varied in relation to their initial composition and in the fitness benefits of receiving cooperation. Cost of cooperation for the actor was always 1 fitness unit. Each panel shows the relative proportions of the two strategies along successive generations in 30 replicates for each combination of parameters (means and standard deviations).

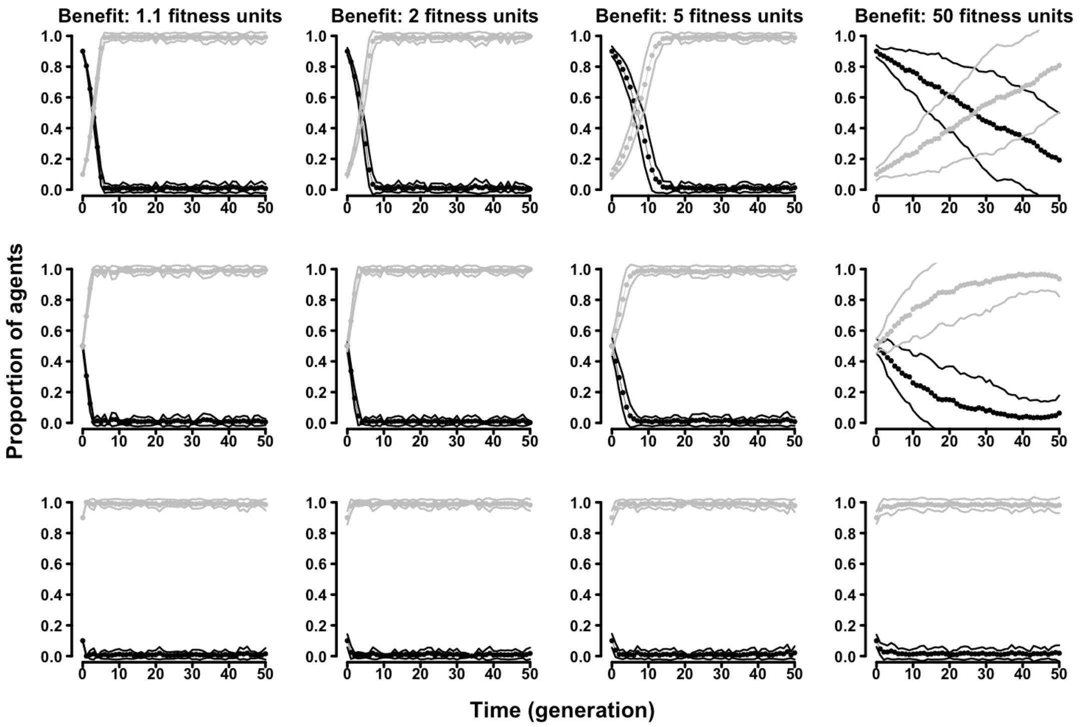

Figure S7. The evolution of cooperation in the "two-strategy" Tag Model. Populations were formed by agents adopting either of two different strategies: symmetry-based cooperation (black dots) and selfish (grey dots). Symmetry-based cooperators could make their choice among 25 randomly selected other agents. Populations varied in relation to their initial composition and in the fitness benefits of receiving cooperation. Cost of cooperation for the actor was always 1 fitness unit. Each panel shows the relative proportions of the two strategies along successive generations in 30 replicates for each combination of parameters (means and standard deviations).

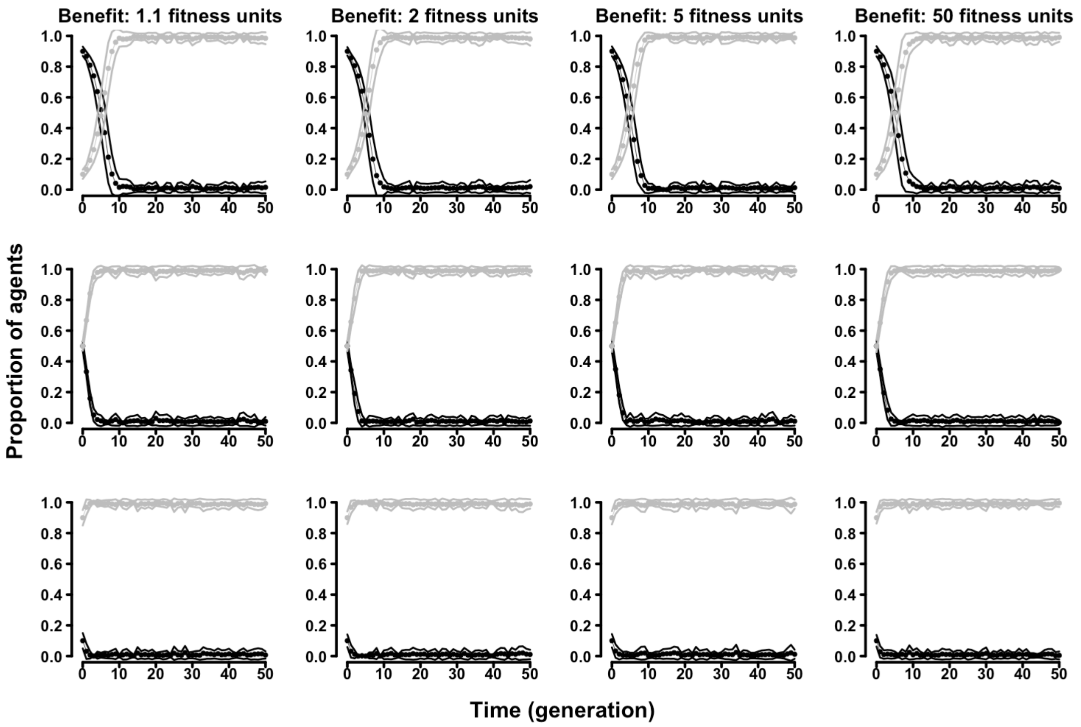

Figure S8. The evolution of cooperation in the "two-strategy" Tag Model. Populations were formed by agents adopting either of two different strategies: symmetry-based cooperation (black dots) and selfish (grey dots). Symmetry-based cooperators could make their choice among all 49 other agents in the population. Populations varied in relation to their initial composition and in the fitness benefits of receiving cooperation. Cost of cooperation for the actor was always 1 fitness unit. Each panel shows the relative proportions of the two strategies along successive generations in 30 replicates for each combination of parameters (means and standard deviations).

Figures S9-S12 show the results of the simulations obtained running the "two-strategy" variant of the evolutionary Proximity Model, where the relative success of two fixed strategies (symmetry-based cooperation and selfish) was evaluated along generations. In the different figures, agents could make their choice among 2, 10, 25 or 49 candidates.

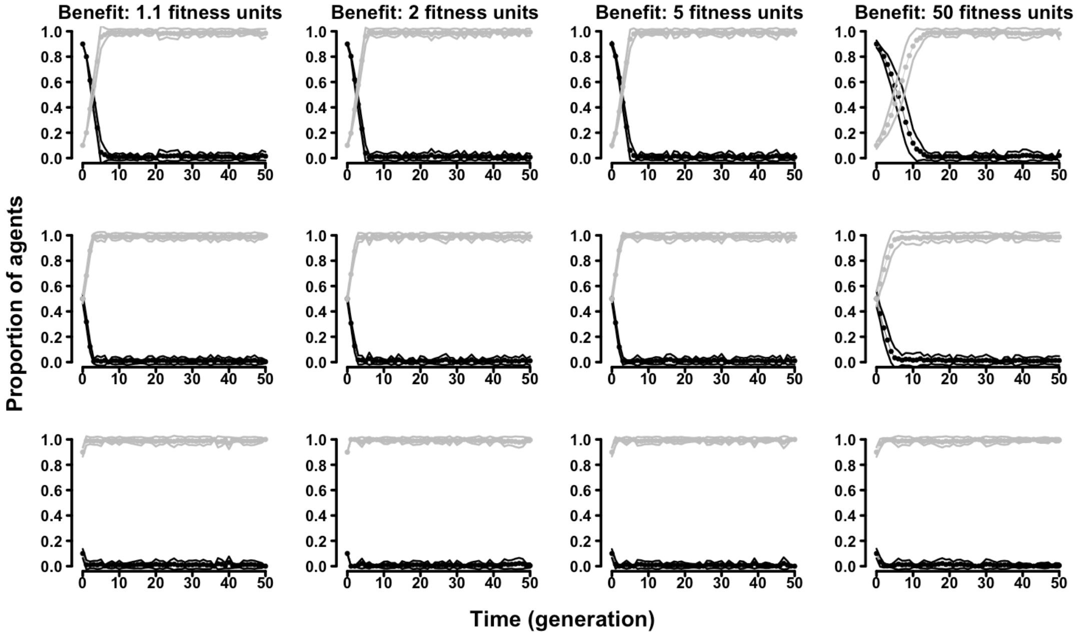

Figure S9. The evolution of cooperation in the "two-strategy" Proximity Model. Populations were formed by agents adopting either of two different strategies: symmetry-based cooperation (black dots) and selfish (grey dots). Symmetry-based cooperators could make their choice among 2 randomly selected other agents. Populations varied in relation to their initial composition and in the fitness benefits of receiving cooperation. Cost of cooperation for the actor was always 1 fitness unit. Each panel shows the relative proportions of the two strategies along successive generations in 30 replicates for each combination of parameters (means and standard deviations).

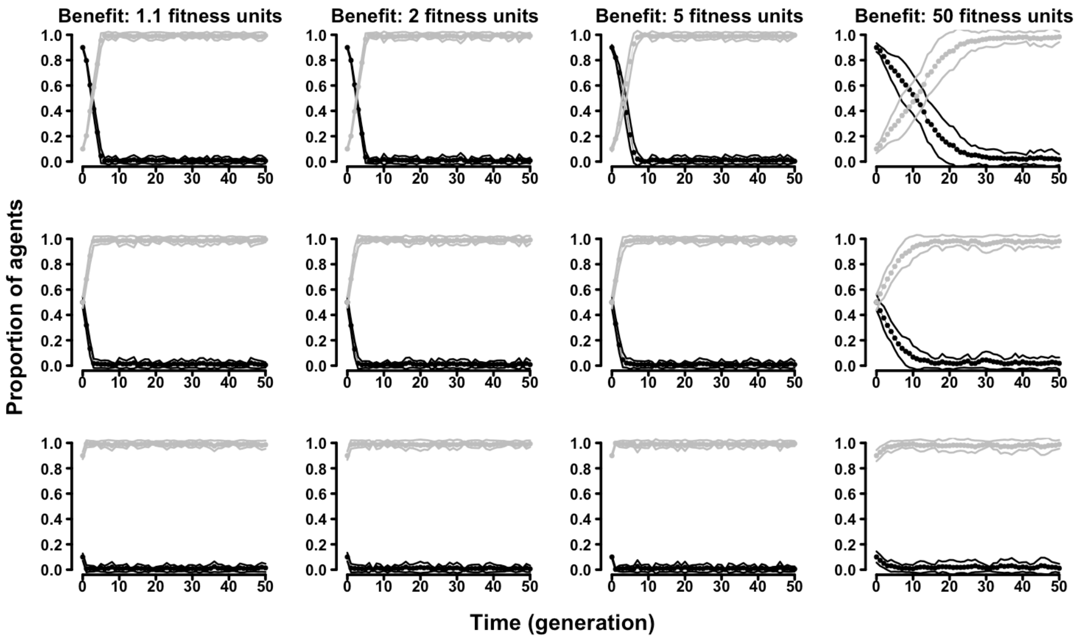

Figure S10. The evolution of cooperation in the "two-strategy" Proximity Model. Populations were formed by agents adopting either of two different strategies: symmetry-based cooperation (black dots) and selfish (grey dots). Symmetry-based cooperators could make their choice among 10 randomly selected other agents. Populations varied in relation to their initial composition and in the fitness benefits of receiving cooperation. Cost of cooperation for the actor was always 1 fitness unit. Each panel shows the relative proportions of the two strategies along successive generations in 30 replicates for each combination of parameters (means and standard deviations).

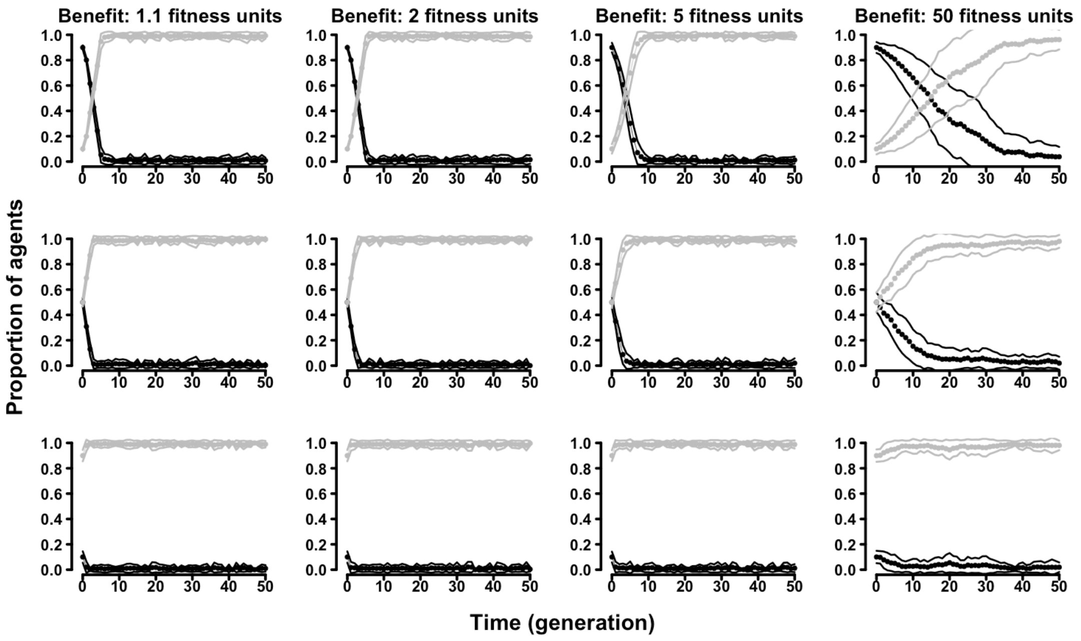

Figure S11. The evolution of cooperation in the "two-strategy" Proximity Model. Populations were formed by agents adopting either of two different strategies: symmetry-based cooperation (black dots) and selfish (grey dots). Symmetry-based cooperators could make their choice among 25 randomly selected other agents. Populations varied in relation to their initial composition and in the fitness benefits of receiving cooperation. Cost of cooperation for the actor was always 1 fitness unit. Each panel shows the relative proportions of the two strategies along successive generations in 30 replicates for each combination of parameters (means and standard deviations).

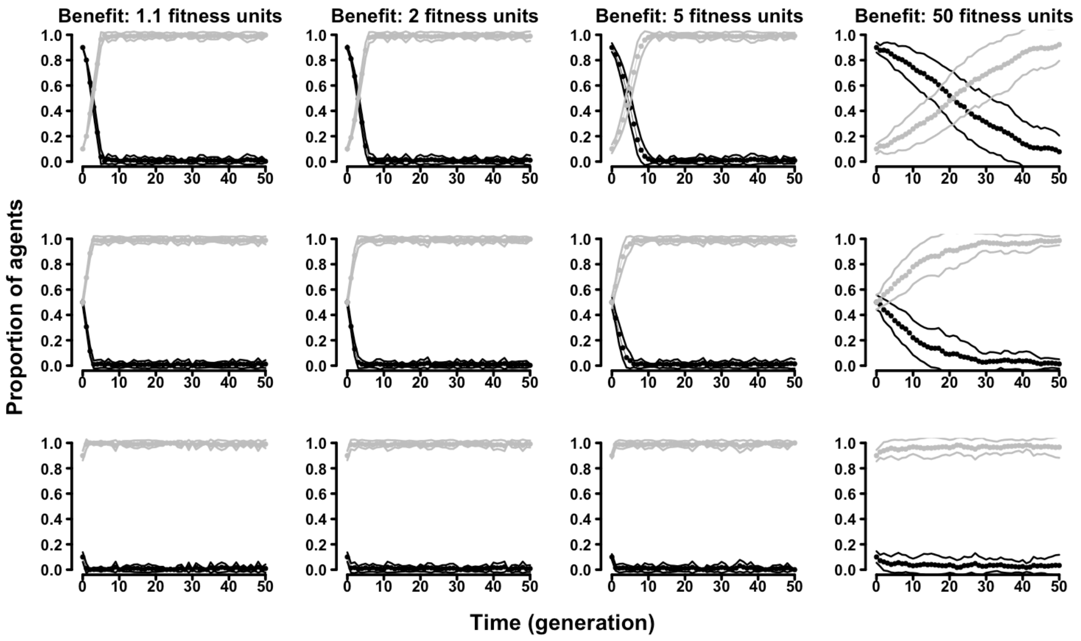

Figure S12. The evolution of cooperation in the "two-strategy" Proximity Model. Populations were formed by agents adopting either of two different strategies: symmetry-based cooperation (black dots) and selfish (grey dots). Symmetry-based cooperators could make their choice among all 49 other agents in the population. Populations varied in relation to their initial composition and in the fitness benefits of receiving cooperation. Cost of cooperation for the actor was always 1 fitness unit. Each panel shows the relative proportions of the two strategies along successive generations in 30 replicates for each combination of parameters (means and standard deviations).

"Continuous probability" models

Figures S13-S16 show the results of the simulations obtained running the "continuous probability" variant of the evolutionary Tag Model. Agents were characterized by an individual probability P of behaving as a symmetry-based cooperator, and changes in P along generations were evaluated. In the different figures, agents could make their choice among 2, 10, 25 or 49 candidates.

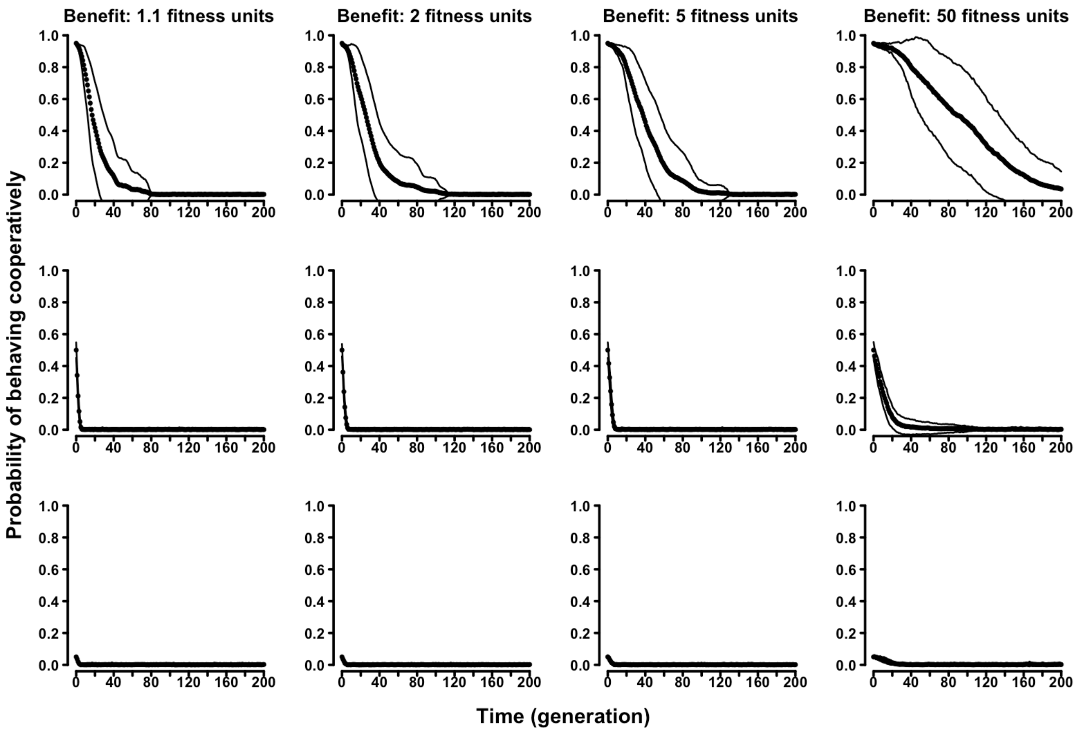

Figure S13. The evolution of cooperation in the "continuous probability" Tag Model. Populations formed by agents varying in their probability of behaving as a symmetry-based cooperator. Choosing cooperators could make their choice among 2 randomly selected other agents. Populations varied in relation to the initial average probability of behaving as a choosing cooperator and in the fitness benefits of receiving cooperation. Cost of cooperation for the actor was always 1 fitness unit. Each panel shows the probability of behaving as a choosing cooperator along successive generations in 30 replicates for each combination of parameters (means and standard deviations).

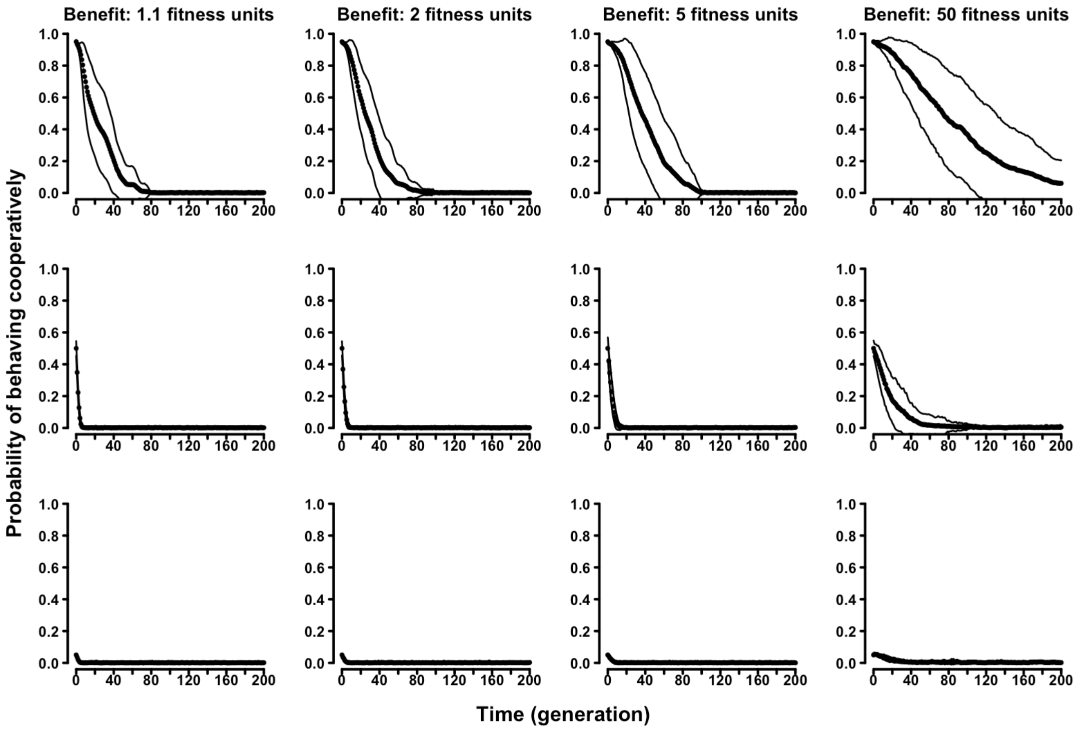

Figure S14. The evolution of cooperation in the "continuous probability" Tag Model. Populations formed by agents varying in their probability of behaving as a symmetry-based cooperator. Choosing cooperators could make their choice among 10 randomly selected other agents. Populations varied in relation to the initial average probability of behaving as a choosing cooperator and in the fitness benefits of receiving cooperation. Cost of cooperation for the actor was always 1 fitness unit. Each panel shows the probability of behaving as a choosing cooperator along successive generations in 30 replicates for each combination of parameters (means and standard deviations).

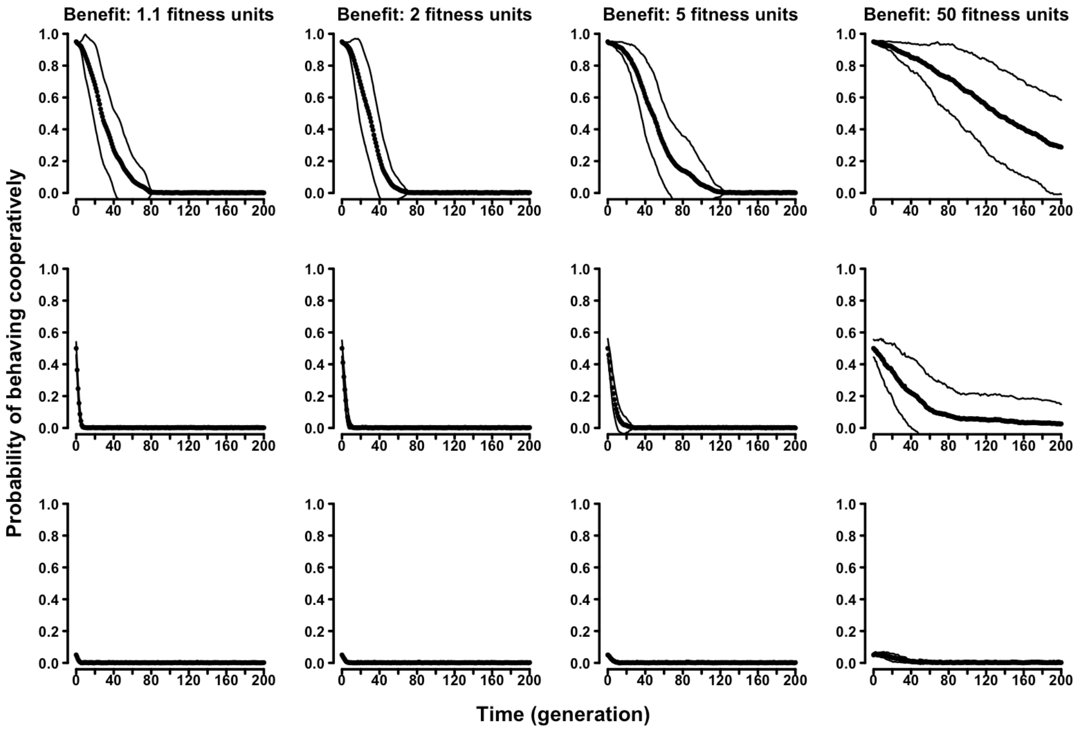

Figure S15. The evolution of cooperation in the "continuous probability" Tag Model. Populations formed by agents varying in their probability of behaving as a symmetry-based cooperator. Choosing cooperators could make their choice among 25 randomly selected other agents. Populations varied in relation to the initial average probability of behaving as a choosing cooperator and in the fitness benefits of receiving cooperation. Cost of cooperation for the actor was always 1 fitness unit. Each panel shows the probability of behaving as a choosing cooperator along successive generations in 30 replicates for each combination of parameters (means and standard deviations).

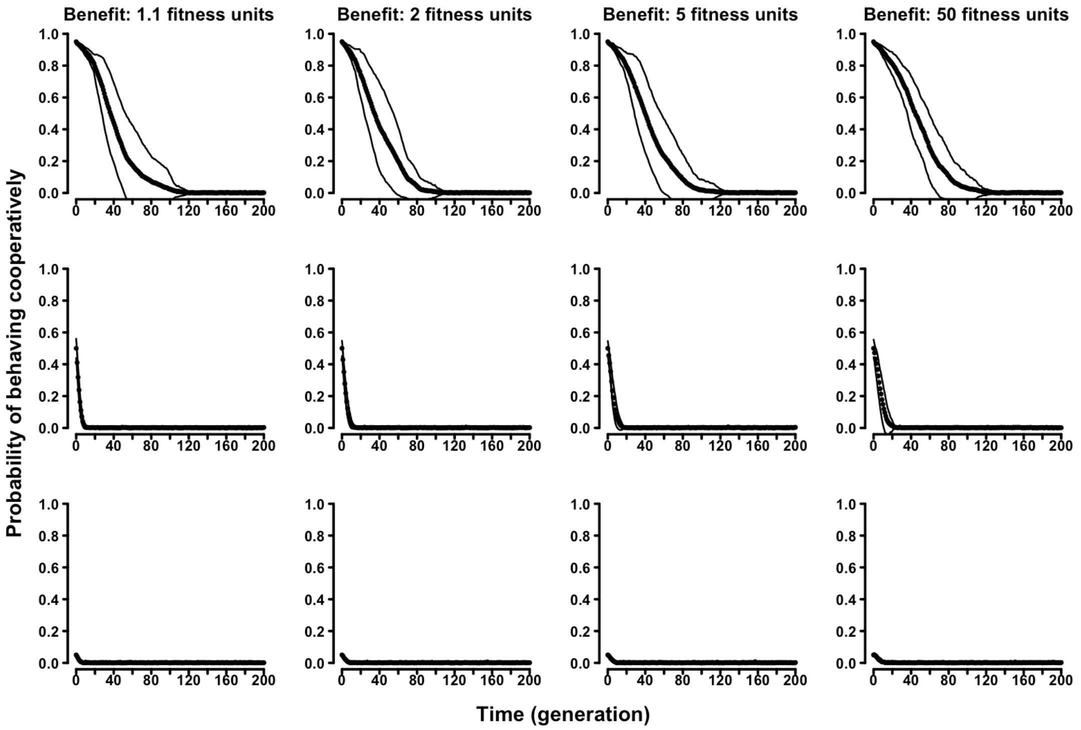

Figure S16. The evolution of cooperation in the "continuous probability" Tag Model. Populations formed by agents varying in their probability of behaving as a symmetry-based cooperator. Choosing cooperators could make their choice among all 49 other agents in the population. Populations varied in relation to the initial average probability of behaving as a choosing cooperator and in the fitness benefits of receiving cooperation. Cost of cooperation for the actor was always 1 fitness unit. Each panel shows the probability of behaving as a choosing cooperator along successive generations in 30 replicates for each combination of parameters (means and standard deviations).

Figures S17-S20 show the results of the simulations obtained running the "continuous probability" variant of the evolutionary Proximity Model. Agents were characterized by an individual probability P of behaving as a symmetry-based cooperator, and changes in P along generations were evaluated. In the different figures, agents could make their choice among 2, 10, 25 or 49 candidates.

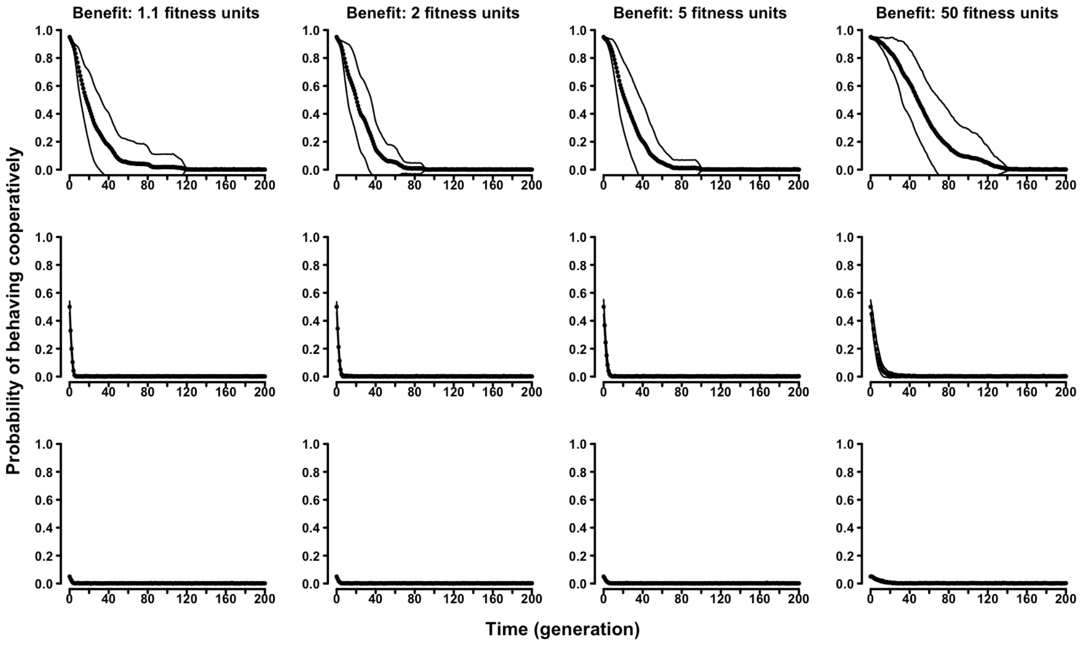

Figure S17. The evolution of cooperation in the "continuous probability" Proximity Model. Populations formed by agents varying in their probability of behaving as a symmetry-based cooperator. Choosing cooperators could make their choice among 2 randomly selected other agents. Populations varied in relation to the initial average probability of behaving as a choosing cooperator and in the fitness benefits of receiving cooperation. Cost of cooperation for the actor was always 1 fitness unit. Each panel shows the probability of behaving as a choosing cooperator along successive generations in 30 replicates for each combination of parameters (means and standard deviations).

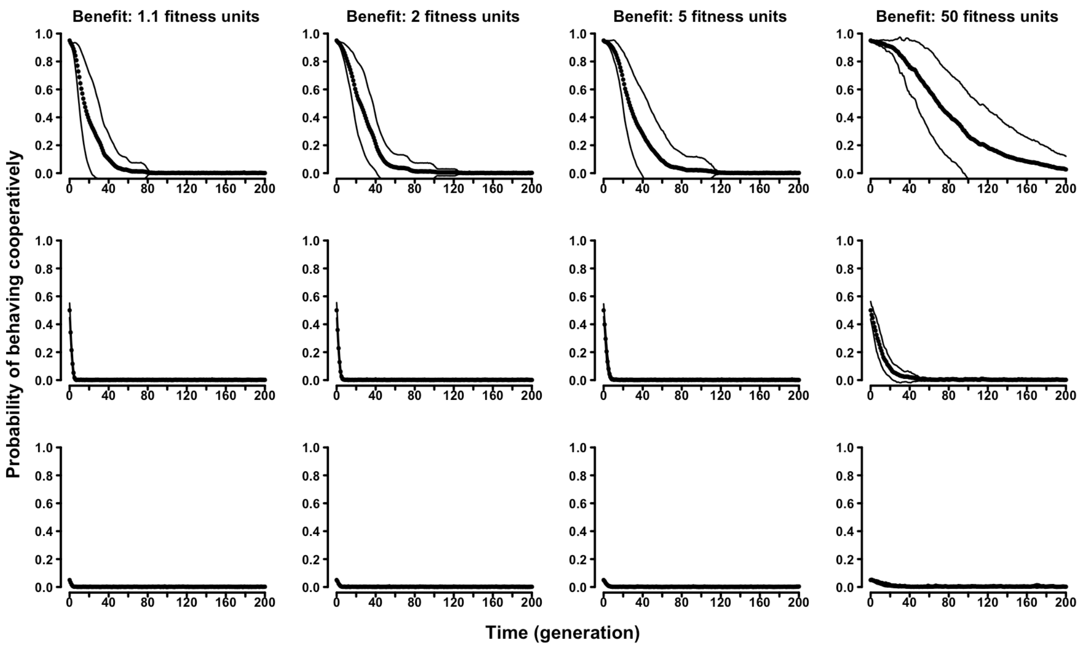

Figure S18. The evolution of cooperation in the "continuous probability" Proximity Model. Populations formed by agents varying in their probability of behaving as a symmetry-based cooperator. Choosing cooperators could make their choice among 10 randomly selected other agents. Populations varied in relation to the initial average probability of behaving as a choosing cooperator and in the fitness benefits of receiving cooperation. Cost of cooperation for the actor was always 1 fitness unit. Each panel shows the probability of behaving as a choosing cooperator along successive generations in 30 replicates for each combination of parameters (means and standard deviations).

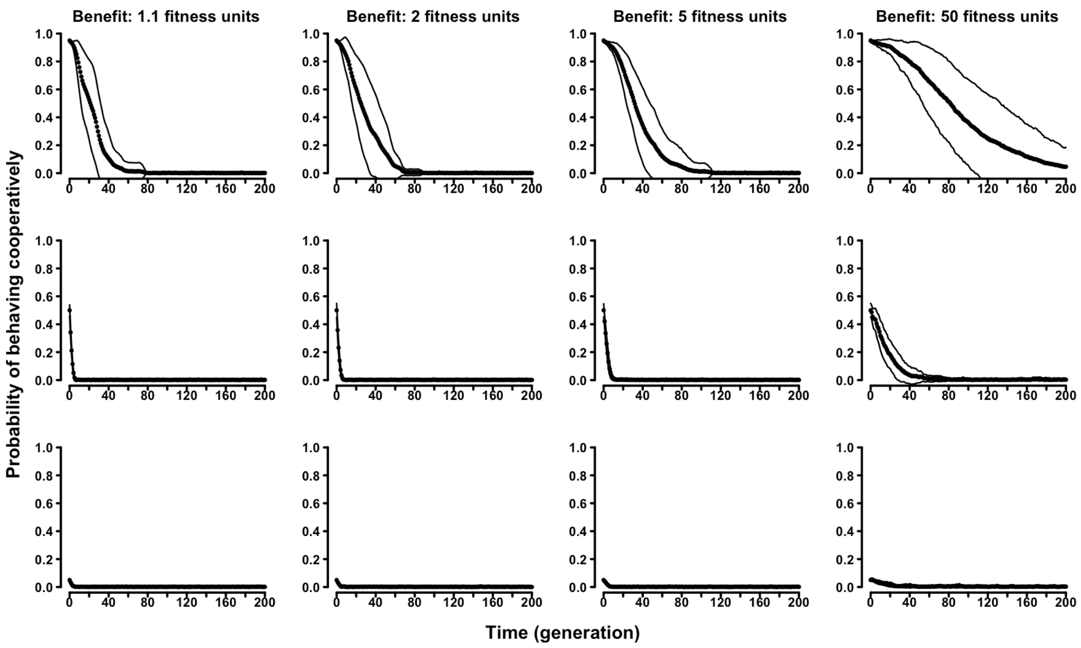

Figure S19. The evolution of cooperation in the "continuous probability" Proximity Model. Populations formed by agents varying in their probability of behaving as a symmetry-based cooperator. Choosing cooperators could make their choice among 25 randomly selected other agents. Populations varied in relation to the initial average probability of behaving as a choosing cooperator and in the fitness benefits of receiving cooperation. Cost of cooperation for the actor was always 1 fitness unit. Each panel shows the probability of behaving as a choosing cooperator along successive generations in 30 replicates for each combination of parameters (means and standard deviations).

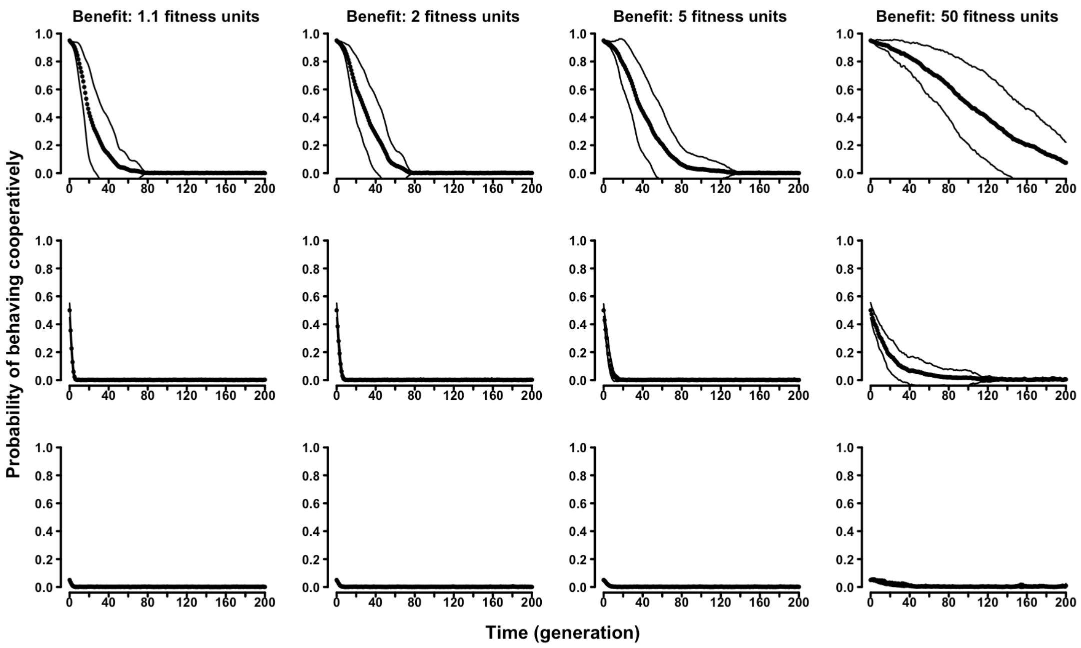

Figure S20. The evolution of cooperation in the "continuous probability" Proximity Model. Populations formed by agents varying in their probability of behaving as a symmetry-based cooperator. Choosing cooperators could make their choice among all 49 other agents in the population. Populations varied in relation to the initial average probability of behaving as a choosing cooperator and in the fitness benefits of receiving cooperation. Cost of cooperation for the actor was always 1 fitness unit. Each panel shows the probability of behaving as a choosing cooperator along successive generations in 30 replicates for each combination of parameters (means and standard deviations).

REFERENCES

Grimm V, Berger U, Bastiansen F, Eliassen S, Ginot V, Giske J, Goss-Custard J, Grand T, Heinz S, Huse G, Huth A, Jepsen JU, Jørgensen C, Mooij WM, Müller B, Pe'er G, Piou C, Railsback SF, Robbins AM, Robbins MM, Rossmanith E, Rüger N, Strand E, Souissi S, Stillman RA, Vabø R, Visser U, DeAngelis DL. 2006. A standard protocol for describing individual-based and agent-based models. *Ecol Model* **198**:115-126.

Pasquaretta C, Levé M, Claidiere N, van de Waal E, Whiten A, MacIntosh AJJ, Pelé M, Bergstrom ML, Borgeaud C, Brosnan SF, Crofoot MC, Fedigan LM, Fichtel C, Hopper LM, Mareno MC, Petit O, Schnoell AV, Polizzi di Sorrentino E, Thierry B, Tiddi B, Sueur C. 2014. Social networks in primates: smart and tolerant species have more efficient networks. *Sci Rep* **4**: 7600.
